# Delayed response to proton beam treatment of hepatocellular carcinoma

**DOI:** 10.1259/bjrcr.20180125

**Published:** 2020-02-12

**Authors:** Chee-Wai Cheng, Mitchell Machtay, Jennifer Dorth, Olga Sergeeva, Hangsheng Xia, chawan manaspon, Hanping wu, Renuka Iyer, Sandra Sexton, Wei Xin, Agata A Exner, Zhenghong Lee

**Affiliations:** 1 Radiation Oncology, University Hospitals Cleveland Medical Center, ClevelandOH, United States; 2 Radiology, Case Western Reserve University, ClevelandOH, United States; 3 Radiology, University Hospitals Cleveland Medical Center, ClevelandOH, United States; 4 Medical Oncology, Rowell Park Cancer Institute, BuffaloNY, United States; 5 Pathology, University Hospitals Cleveland Medical Center, ClevelandOH, United States

## Abstract

Hepatocellular carcinoma (HCC) has become one of the leading causes of cancer death worldwide. There has been anecdotal report regarding the effectiveness of proton beam treatment for HCC. In this pre-clinical investigation, the woodchuck model of viral hepatitis infection-induced HCC was used for proton beam treatment experiment. The radiopaque fiducial markers that are biodegradable were injected around the tumor under ultrasound guidance to facilitate positioning in sequential treatments. An α cradle mode was used to ensure reproducibility of animal positioning on the treatment couch. A CT scan was performed first for contouring by a radiation oncologist. The CT data set with contours was then exported for dose planning. Three fractionations, each 750 CcGyE, were applied every other day with a Mevion S250 passive scattering proton therapy system. Multiphase contrast-enhanced CT scans were performed after the treatment and at later times for follow-ups. 3 weeks post-treatment, shrinking of the HCC nodule was detected and constituted to a partial response (30% reduction along the long axis). By week nine after treatment, the nodule disappeared during the arterial phase of multiphase contrast-enhanced CT scan. Pathological evaluation corroborated with this imaging response. A delayed, but complete imaging response to proton beam treatment applied to HCC was achieved with this unique and clinically relevant animal model of HCC.

## Investigation

Hepatocellular carcinoma (HCC) is a primary malignancy of the liver. Chronic infections with hepatitis B virus (HBV) and hepatitis C virus (HCV) are among the most important risk factors for the development of HCC in humans. It is one of the leading causes of cancer deaths worldwide, with over 600,000 people affected in 2015.^1^ Clinical indications of radiotherapy include: large unresectable HCC; relieving portal vein thrombosis and obstructive jaundice; failure of prior transarterial chemoembolization (TACE); or as part of combined chemoradiaiton treatment.^2^ The role of the conventional external beam radiation therapy (EBRT) for HCC had been limited due to the low radiation tolerance of normal liver to therapeutic doses. However, advances in radiation therapy and imaging technologies have led to increasing use of radiation therapy for HCC, mostly with stereotactic body radiotherapy (SBRT)—high dose per fraction and very conformal radiotherapy.^3^ For example, the current RTOG-1112 study is ongoing, which is a randomized trial of systemic therapy ± SBRT for the patients with advanced HCC to determine whether SBRT improves survival in these patients. The main issue with SBRT is the mean liver dose, which is most predictive of worsening liver function after SBRT.^4^ On the other hand, proton beam therapy offers dosimetric advantage of spread out Bragg Peak (SOBP) delivering high doses to the tumor with limiting normal tissue toxicity distal to the beam.^5^ Therefore, the use of protons can potentially decrease the mean liver dose and preserve liver function. Clinical evidence regarding the effectiveness of proton beam treatment for HCC has been discussed recently.^6^

As a clinically relevant animal model, the eastern woodchuck (*Marmota monax*) develops HCC after chronic viral hepatitis infection as it harbors a DNA virus—the woodchuck hepatitis virus (WHV), 80% homologous to human HBV. Similar to HBV, WHV infects the liver to cause acute and chronic hepatitis. Chronic WHV infection in woodchucks usually leads to development of HCC within the first 2–4 (human) years of their life (one human year is approximately 5.721 woodchuck years). Chronic WHV viral infection-induced HCC in woodchucks has shown similar pathology and natural history with human HCC originated from chronic HBV infection, even though it lacks the clinical manifestation of cirrhosis,^7–9^ which is also absent in about 10% human HCCs from chronic HBV infection in United States alone^10^ although as many as 20% HCCs involve non-cirrhotic livers.^11^ While the etiology of HCC is very complex, with many factors affecting disease course and patient prognosis, there are no published reports on radiation treatment outcome for a specific etiology. In this study, we used this woodchuck model of WHV infection-induced HCC to test the efficacy of passive scattering proton beams on the treatment of HCC with high dose fractionation, which have not been reported before. All experiments were approved by Institutional Animal Care and Use Committee (protocol #2014–0085) and Radiation Safety Committee (Protocol#773).

## Treatment

**Figure 1.**
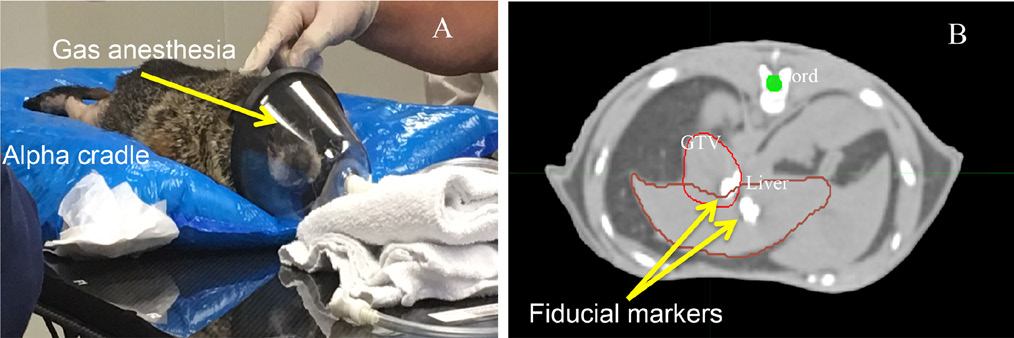
Experimental setup: (A) Woodchuck immobilized in the alpha-cradle mold. Gas anesthesia is administered throughout the treatment. (B) A CT slice to show the implanted fiducial markers and the contours outlined by the physician.

**Figure 2.**
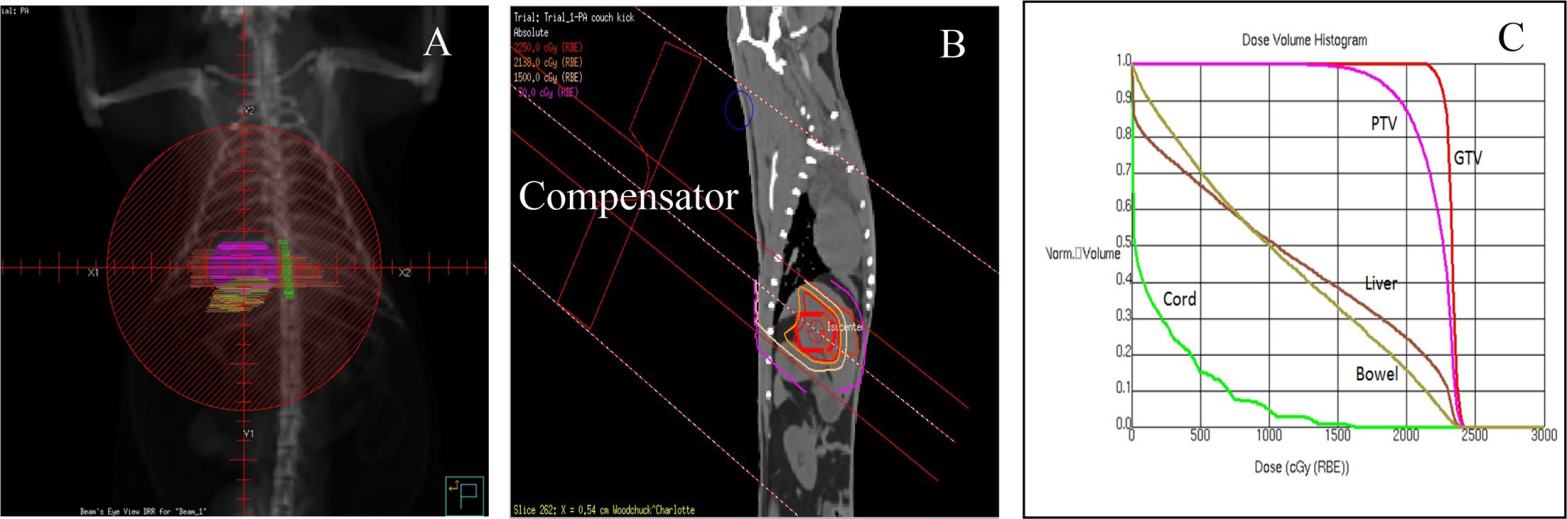
Treatment planning: (A) Beams-eye-view showing the design of the brass aperture to conform to the shape of the tumor (purple) while shielding partially the liver (brown) and bowel (yellow). (B) Dose distribution in the sagittal plane through the isocenter. (C) Dose–volume histogram of the GTV, PTV and the organs-at-risk. GTV, gross tumor volume; PTV, planning target volume.

Radiopaque fiducial markers that are biodegradable were created by co-dissolving poly(lactide-co-glycolide) polymer and lyophilized iodinated contrast agent, Ioversol^®^, in a water-miscible organic solvent (n-methyl-pyrrolidone). Markers were injected around the tumor under ultrasound guidance to facilitate tumor localization during treatment. An α cradle mold (Smithers Medical Products, Inc., N Canton, OH) was custom-made to immobilize the animal in prone position for the CT scanning. The same mold will be used to reproduce the positioning of the woodchuck on the treatment couch (Figure 1A). The animal, under gas anesthesia, was first subjected to a CT scan, which was exported to the imaging software MIM (Cleveland, OH) for contouring of the tumor by a radiation oncologist. An example of the tumor, the liver and the spinal cord outlined in a CT image is shown in Figure 1B. The locations of the fiducial markers can be seen clearly in Figure 1B. The CT data set was then exported to the Pinnacle treatment planning system (Philips Healthcare, Andover, MA) for dose planning using the beam data of a Mevion S250 passive scattering proton therapy system (Littleton, MA). Because of the location of the tumor relative to liver, a three-dimensional conformal proton plan with a single non-coplanar field was generated so that at least 97% of the gross tumor volume (GTV) received at least 98% of the prescribed dose (750 CcGE/fx). Due to the high dose fraction, the animal was treated every other day within 1 week: Monday, Wednesday and Friday. Prior to each treatment, the animal was set up on the treatment couch in the α cradle under gas anesthesia. Image guidance was performed with the Mevion Verity X-ray system to reposition the animal as in CT scan with the aid of the fiducial markers and the anatomy. The beams-eye-view, the dose distribution in the sagittal plane through the isocenter and the dose–volume histogram (DVH) of the plan are shown in Figure 2, in which the margin between the GTV and the planning target volume (PTV) was estimated to be 6–7 mm.

**Figure 3.**
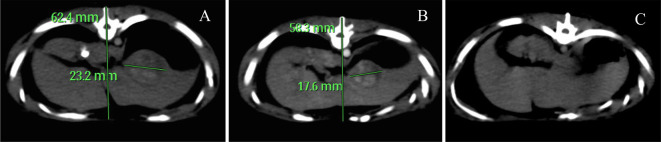
Tracking the response to proton beam treatment with multiphase contrast-enhanced CT: (A) At the end of treatment, (B) 3 weeks after treatment, (C) 9 weeks after treatment.

Right after the proton beam treatment, the animal was transported to a clinical positron emission tomography (PET)/CT scanner nearby and positioned for a whole body 5 min static PET image acquisition starting at 15 min post proton beam assuming C-11 as the endogenous positron emitting radionuclide generated by along the path of the proton beam. There are a few testing units existing with a built-in PET component orthogonal to the treatment beam,^12^ which allowed simultaneous PET imaging of the distribution positrons generated by the proton-beam for a quick assessment of the delivered dose. Here, the residue radioactivity at 15–20 min of after the beam was imaged only to confirm the residue of beam summation. Multiphase contrast-enhanced (MPCE) CT scans were performed right after the treatment, and 3 and 9 weeks post proton therapy as follow-ups (Figure 3).

## Outcome

**Figure 4.**
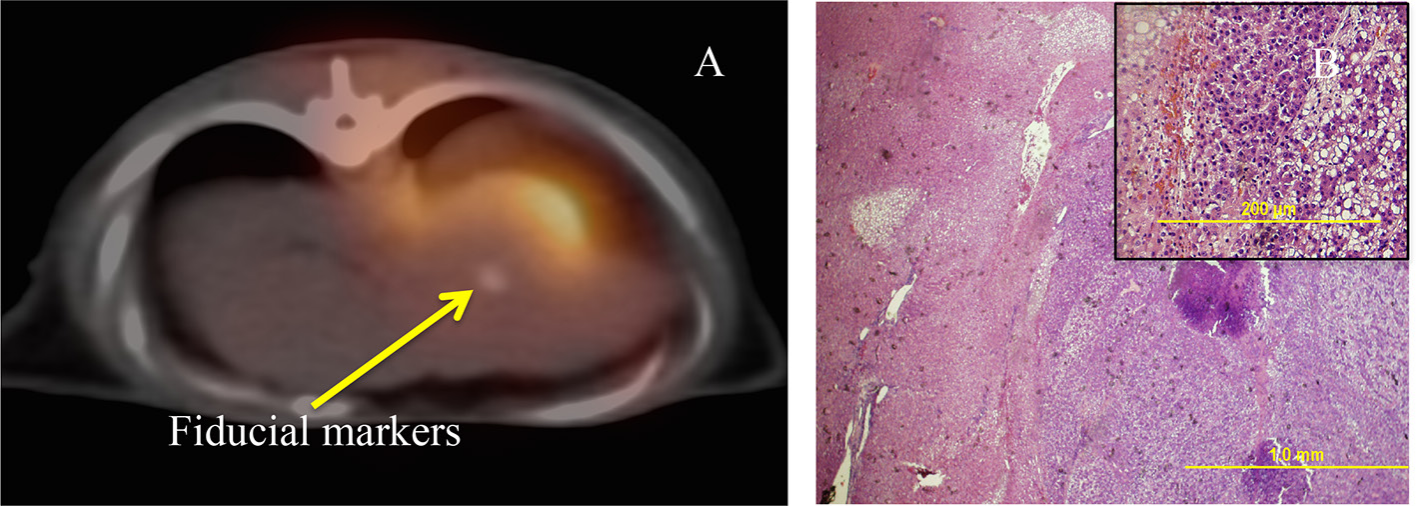
(A) 5 min static scan starting at 15 min after the proton beam treatment. In this slightly “delayed” PET scan (usually 2 min post-treatment is recommended for confirming the beam), the radioactivity is retained mostly in the outer side-of the tumor while the intensity at the beam entrance in the back (skins and bone) subsided. (B) H&E staining of the harvested liver tissue from the transition zone along the path of proton beam. PET, positron emission tomography.

Figure 4A is the PET/CT overlay showing the radioactivity in the area being targeted. By 15–20 min post-proton beam, most of the remaining radionuclides generated by the proton-beam are C-11 as the concurrent O-15 that was also generated by the proton-beam was either decayed or washed away. From Figure 3, shrinking of the HCC nodule was detected 3 weeks post-treatment and constituted a partial response (30% reduction in the long axis). By Week 9, the nodules disappeared in the arterial phase of MPCE CT scan, indicating a complete imaging response. Pathological evaluation (Figure 4B) corroborated with this imaging response. In addition, the animal had no blood in the urine and maintained its weight for 10 weeks after the treatment demonstrating little gastrointestinal (GI) toxicity.

## Discussion

The woodchuck model has been used for studies of biological properties of HBV.^7^ In the present study, delayed response to the high dose fractionation typical that of SBRT but delivered with a passive scattering proton beam was observed with this spontaneous animal model of chronic viral hepatitis infection-induced HCC in the woodchuck, with a complete imaging response by nine weeks post-treatment.

There are multiple etiology factors affecting HCC, all of which have a direct impact on the characteristics of the disease and its response to treatment. The positive outcome in the present case study seems to suggest that SBRT-like high dose fractionation may be effective for HBV infection-induced HCC without the presence of cirrhosis, which is modeled by the chronic viral hepatitis infection-induced HCC in the woodchucks that present some degree of fibrosis, but not the clinical phenotype of cirrhosis as discussed above. Prospective clinical studies are needed to validate the finding reported here. Noticeably, HBV infection-associated liver cancers seem to have a better prognosis in the non-fibrotic or minimally fibrotic patients.^13^ Finally, while MPCE CT scans can be reliably used to track such treatment response, PET images taken right after each proton-beam without injection of radiotracer(s) can also be investigated further to evaluate their utility for tracking response to proton beam treatment.

## Learning points

1. There have been pre-clinical setups/tests of (mini)-proton beam treatment on mouse tumor models, mostly xenografts. In this study, a mid-sized and clinically relevant spontaneous woodchuck model of chronic viral hepatitis-infection induced HCC was successfully tested on the clinical proton-beam treatment unit.
2. The case presented illustrates a complete imaging response of a subtype of HCC (chronic viral hepatitis infection-induced without cirrhosis) to proton beam treatment with a high dose fractionation without visible GI toxicity. The conversion of biologic equivalent dose of what was delivered to the animal is higher than the current clinical treatment regimens.
3. Future clinical studies will validate the pre-clinical finding for the subpatient population mentioned above. The uniqueness of proton beam treatment applied to this subpopulation of HCC patients is to take the advantage of the effectiveness of SBRT while preserve liver function. Additionally, one will need to carefully tracking weight as a surrogate for GI symptoms even though this was not an issue for the woodchuck model. Furthermore, the observed imaging response was delayed (Note: nine human weeks are a much longer time span for the woodchucks). Proper timing of the follow-up for human patients will also need to be established.
